# Nitric Oxide Metabolic Pathway in Drought-Stressed Nodules of Faba Bean (*Vicia faba* L.)

**DOI:** 10.3390/ijms232113057

**Published:** 2022-10-27

**Authors:** Chaima Chammakhi, Alexandre Boscari, Marie Pacoud, Grégoire Aubert, Haythem Mhadhbi, Renaud Brouquisse

**Affiliations:** 1 Sophia Agrobiotech Institute, INRAE 1355, CNRS 7254, Côte d’Azur University, 06903 Sophia Antipolis, France; 2 Laboratory of Legumes and Sustainable Agrosystems, Biotechnology Center of Borj-Cedria, Hammam-Lif 2050, Tunisia; 3 National Agronomic Institute of Tunisia, University of Carthage, Tunis 1082, Tunisia; 4 Agroecology, INRAE, Agro Institute, Bourgogne Franche-Comté University, 21065 Dijon, France

**Keywords:** drought stress, hypoxia, faba bean, legume, nitric oxide, nitrogen-fixing symbiosis, phytoglobin no respiration

## Abstract

Drought is an environmental stress that strongly impacts plants. It affects all stages of growth and induces profound disturbances that influence all cellular functions. Legumes can establish a symbiosis with Rhizobium-type bacteria, whose function is to fix atmospheric nitrogen in organs called nodules and to meet plant nitrogen needs. Symbiotic nitrogen fixation (SNF) is particularly sensitive to drought. We raised the hypothesis that, in drought-stressed nodules, SNF inhibition is partly correlated to hypoxia resulting from nodule structure compaction and an increased O_2_ diffusion barrier, and that the nodule energy regeneration involves phytoglobin–nitric oxide (Pgb–NO) respiration. To test this hypothesis, we subjected faba bean (*Vicia faba* L.) plants nodulated with a *Rhizobium laguerreae* strain to either drought or osmotic stress. We monitored the N_2_-fixation activity, the energy state (ATP/ADP ratio), the expression of hypoxia marker genes (alcohol dehydrogenase and alanine aminotransferase), and the functioning of the Pgb–NO respiration in the nodules. The collected data confirmed our hypothesis and showed that (1) drought-stressed nodules were subject to more intense hypoxia than control nodules and (2) NO production increased and contributed via Pgb–NO respiration to the maintenance of the energy state of drought-stressed nodules.

## 1. Introduction

Fresh water is a fragile and increasingly scarce resource. Throughout the world, the decrease in rainfall, the increase in withdrawals for agricultural and non-agricultural uses, and global warming have resulted in a drop in groundwater tables associated with increasing agricultural drought [1,2]. Drought is one of the stresses, if not the stress, that most strongly impacts plant growth. It is responsible for 50–70% of crop productivity and yield losses [3,4,5]. Due to the importance of water in biological processes, drought stress (DS) affects all stages of plant growth, from germination to maturity, and induces profound disturbances that affect virtually all cellular functions [6,7,8,9]. DS leads to tissue dehydration and to a drop in water potential, which results in a reduction in cell expansion, cell wall synthesis, and organ growth [10]. One of the most dramatic effects of DS is the inhibition of leaf photosynthesis via stomatal closure and a subsequent decrease in the CO_2_ flux in mesophyll cells, the inhibition of ribulose-1,5-bisphosphate carboxylase/oxygenase (rubisco) activity, a reduction in chlorophyll content, and a decrease in the photosynthesis rate [11,12,13]. DS also has many effects on respiration, water and ion transport, the assimilation of mineral nutrients, the overproduction of reactive oxygen species (ROS), enzyme activity, protein synthesis, and nitrogen fixation [7,9,14,15]. To cope with DS, plants set up stress response mechanisms such as the production of reactive oxygen and nitrogen species (H_2_O_2_, ·OH, O_2_^−^, and NO); the synthesis of hormones (ethylene and abscissic acid) and osmoprotective solutes (oligosaccharides, polyols, amino acids, and ammonium compounds); and molecular adaptations that reduce the impact of water deprivation on cellular functioning, water status, and plant growth [8,16,17].

Legumes have the ability to establish a symbiosis with soil bacteria of the Rhizobium type, whose function is to reduce atmospheric nitrogen (N_2_) to ammonia (NH_3_) inside the bacteria and then transfer NH_3_ to the plant to meet its nitrogen needs [18]. This N_2_-fixation process is carried out within a new organ, the nodule, inside which the plant hosts and nourishes the bacteria [19]. For crop legumes, symbiotic N_2_ fixation provides the plant with the nitrogen it needs without resorting to the use of nitrogen fertilizers. To understand the behavior of crop legumes during environmental stresses and to propose appropriate breeding strategies and agronomic practices, the effects of DS in legumes have been widely investigated in recent years [6,7,14,20]. Symbiotic N_2_ fixation (SNF) is a function particularly sensitive to DS. Thus, both infection, nodule development, and nodule function are impaired by DS [6,21,22], and in nodulated soybean plants, DS was shown to inhibit the SNF process before photosynthesis [23]. Several hypotheses, which are not mutually exclusive, have been proposed to explain the decline in SNF during drought [6,24]: (1) the local carbon-based feedback inhibition of SNF linked to the inhibition of sucrose synthase and subsequent sucrose accumulation and organic acid depletion in nodules [25,26,27]; (2) local nitrogen-based feedback regulation linked to the restriction on the export of nitrogen compounds in nodules and their inhibitory action against N_2_ fixation [24,28,29,30]; (3) nitrogen-based systemic regulation linked to the inhibition of SNF by phloem-delivered nitrogen compounds [31,32]; and (4) a decline in oxygen (O_2_) availability for bacteroid respiration linked to an increase in nodular O_2_ diffusion resistance [23,33].

**Figure 1:**
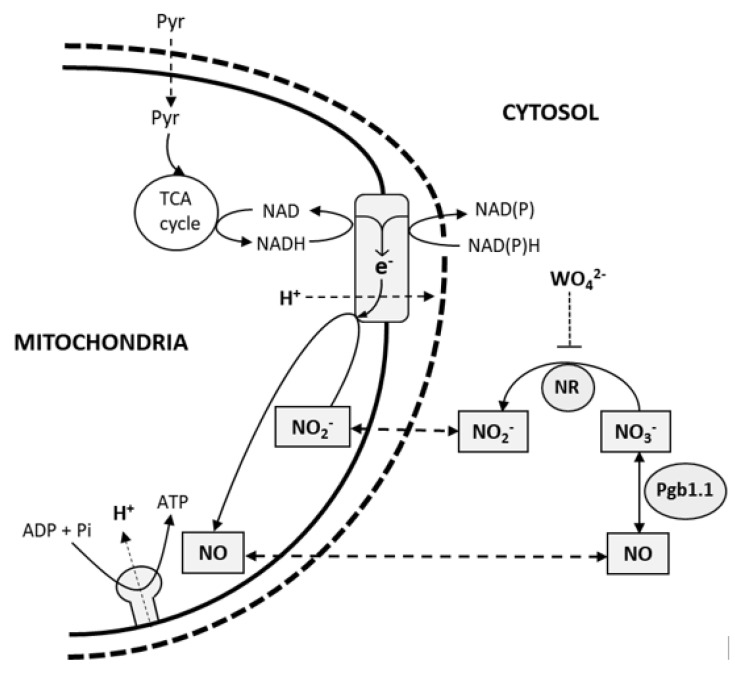
Schematic representation of phytoglobin (Pgb)-NO respiration in hypoxic roots and nodules. Inside mitochondria, pyruvate is oxidized through the tricarboxylic acid (TCA) cycle to generate NADH. Mitochondrial internal dehydrogenase and external dehydrogenases, respectively, oxidize matricial and cytosolic NADH and NADPH. Electrons are transferred to either alternative oxidase or cytochrome oxidase. Nitrite (NO_2_^−^) is reduced into NO at both alternative oxidase and cytochrome oxidase sites. NO diffuses into the cytosol, where it is oxidized into nitrate (NO_3_^−^) by phytoglobins (Pgb1.1). Nitrate reductase (NR) reduces NO_3_^−^ into NO_2_^−^, which is transported into the mitochondria. ATP is synthesized due to the trans-membrane electrochemical gradient generated by proton (H^+^) pumping at the different sites of the electron transfer chain. Tungstate (WO_4_^=^) inhibits Pgb–NO respiration via NR inhibition. Pyr, pyruvate.

Broad bean (*Vicia faba* L.) nodules submitted to DS [33] showed that the decline in SNF was correlated to strong structural changes (a reduction in cortical air spaces, the deformation of cortical cell walls, and the compaction of cortical cell layers) and that optimal pO_2_ for N_2_-fixation activity shifted from 40 kPa for well-watered nodules to 60 kPa for water-deprived nodules. The authors’ conclusion was that N_2_-fixation activity was partly limited by an increase in the nodule O_2_ diffusion barrier resulting from drought-induced nodule cell packing. Similar effects have been observed in soybean, common bean, and alfalfa nodules submitted to salt stress. Indeed, nodule exposure to salinity also resulted in an increase in the nodule O_2_ barrier and a rapid decrease in N_2_-fixation activity, and this decrease was compensated for by raising the pO_2_ [34,35,36,37]. These observations suggested that under salt stress, legume nodules face hypoxia, which limits the SNF. This hypothesis was recently reinforced by the authors of [38], who showed that under salt stress a hypoxia-associated respiration process called phytoglobin (Pgb)–nitric oxide (NO) respiration (Pgb–NO respiration) is induced, which participates in the maintenance of the root and nodule energy state. In the root and/or nodule cells of many plants submitted to hypoxia (*Nicotiana tabacum*, *Hordeum vulgare*, *Oriza sativa, Medicago sativa*, and *M. truncatula*), Pgb–NO respiration was shown to occur, allowing cell energy status retention [39,40,41,42,43]. Pgb–NO respiration (Figure 1) involves: (1) the reduction of NO_3_^−^ to NO_2_^−^ by nitrate reductase (NR); (2) the translocation of NO_2_^−^ from the cytosol to the mitochondrial matrix; (3) the reduction of NO_2_^−^ to NO via the mitochondrial electron transfer chain (ETC), allowing ATP regeneration; and finally (4) the passive diffusion of NO to the cytosol, where it is oxidized back to NO_3_^−^ by Pgb1.1 [43,44,45]. Salinity and drought exhibit a great degree of similarity in their physiological, biochemical, molecular, and genetic effects [46]. Thus, considering the similarity of salinity and drought effects on nodule structure and SNF [23,33,34,37], we hypothesized that under DS legume nodules also face hypoxia, that hypoxia limits SNF, and that Pgb–NO respiration could be involved in maintaining nodule energy status.

To test this hypothesis, we subjected faba bean (*V. faba* L.) plants, nodulated with a *Rhizobium laguerreae* strain, to DS and monitored the N_2_-fixation activity; the energetic state; the expression level of hypoxia marker genes (alanine aminotransferase (*VfAlaAT*) and alcohol dehydrogenase (*VfADH*)); and the functioning of Pgb–NO respiration (NO production; the expression of *VfPgb1.1*, *VfNR1,* and *VfNR2* genes; and the NR activity) in the nodules. Using PEG 6000 stress to mimic DS, we also analyzed the nodule energetic state, NO production, and marker gene expression in the presence or absence of tungstate, a Pgb–NO respiration inhibitor. Based on our results, we discuss the occurrence of hypoxic stress in the nodules and the involvement of Pgb–NO respiration in the energetic state of faba bean nodules under DS.

## 2. Results

### 2.1. Drought Stress Affects Nodule Nitrogen-Fixing Capacity and Energy State and Induces Hypoxia Marker Gene Expression

**Figure 2:**
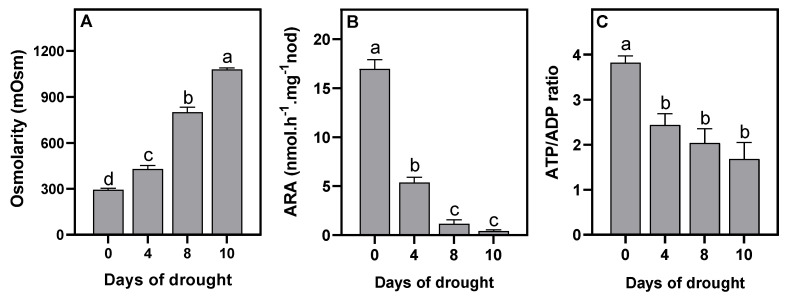
Effects of water deprivation on *Vicia faba* nodule osmolarity (**A**), ARA (**B**), and ATP/ADP ratio (**C**). Data are means ± SD of three biological replicates. Letters above the bars represent significant differences according to one-way ANOVA at *p* < 0.05 (Tukey’s multi-comparison test). ARA, acetylene-reducing activity.

The effects of 10-day-long DS were analyzed in mature nodules of faba bean (*Vicia faba* L., var Bachar) in symbiosis with the *Rhizobium laguerreae* FB206 reference strain tolerant to water stress [47]. As reported in Figure 2A, the osmotic concentration of the nodules was 290 ± 20, which remained constant in the well-watered plants for the duration of the experiment (data not shown). Under DS, the osmolarity increased by about 40% after 4 days of water deprivation and was four-fold increased to reach about 1080 ± 80 mOsm after 10 days. The N-fixation capacity, as measured by ARA, was strongly affected by DS, with 66%, 93%, and >98% inhibition after 4, 8, and 10 days, respectively (Figure 2B), confirming the high sensitivity of SNF to DS [6,24]. Over the course of the DS, the color of the nodules changed from pinky white at 0 and 4 days to brown at 8 days, and then to glassy black at 10 days (data not shown), indicating that after 10 days the tissues were strongly degraded. On the other hand, the analysis of the energy state of the nodules, assessed through the ATP/ADP ratio (Figure 2C), showed that it was clearly affected by DS. From a value of 3.8 ± 0.3 in the control conditions, it decreased to 2.4 ± 0.3 after 4 days, reaching 1.7 ± 0.4 in nodules after 10 days of DS. Drought therefore led to a drop in SNF capacity and to a decrease in the energy state of the nodules.

**Figure 3:**
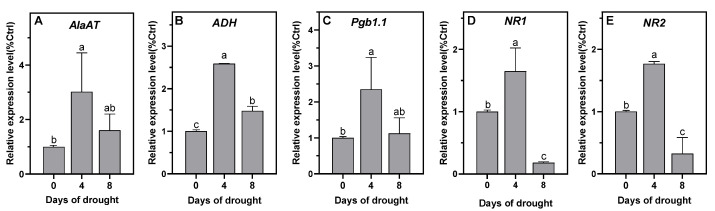
Effects of water deprivation on the relative expression level of Vicia faba nodule genes: alanine aminotransferase (**A**), alcohol dehydrogenase (**B**), phytoglobin1.1 (**C**), nitrate reductase 1 (**D**), and nitrate reductase 2 (**E**). The relative expression of each gene is expressed as the percentage of the control (T = 0) values. Data are means ± SD of three biological replicates. Letters above the bars represent significant differences according to one-way ANOVA at *p* < 0.05 (Tukey’s multi-comparison test). AlaAT, alanine aminotransferase; ADH, alcohol dehydrogenase; Pbg1.1, phytoglobin 1.1; NR1, nitrate reductase 1; NR2, nitrate reductase 2.

In the present work, we hypothesized that the decline in nitrogen-fixation capacity could be partly related to an increase in the DS-induced oxygen barrier, the reinforcement of the hypoxia that prevails in the nodules, and a subsequent decrease in ATP regeneration. Thus, we sought first to monitor the expression of hypoxia marker genes in the plants. Two genes known to be induced in response to hypoxia, i.e., alcohol dehydrogenase (*ADH*) and mitochondrial alanine aminotransferase (*AlaAT*) [48,49], were chosen to test the hypothesis of DS-induced hypoxia in the nodules. After 4 days of DS, the expression of both genes increased by 2.5–3 times in the nodules (Figure 3A,B) and then decreased, which indicated that a transient reinforcement of hypoxia was perceived by the nodule cells during DS. After 10 days of DS, the RNA quality was insufficient and did not allow the tracking of gene expression. This raised the question of the existence of Pgb–NO respiration in faba bean nodules and its potential role in ATP regeneration during DS.

### 2.2. NO Production and Pgb–NO Respiration Are Induced under Drought Stress

**Figure 4:**
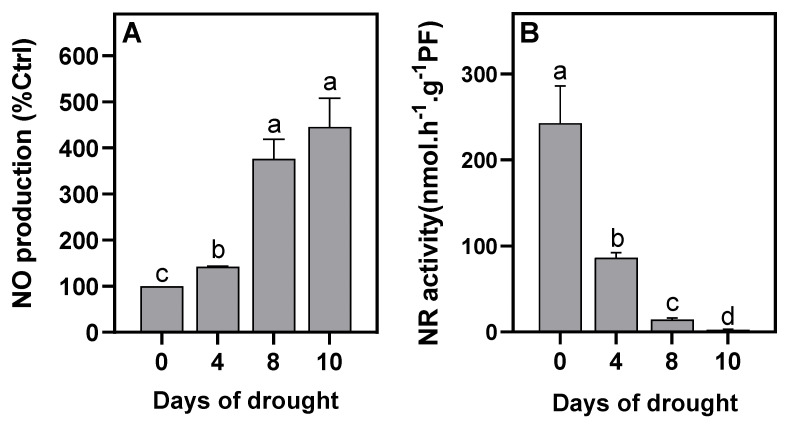
Effects of water deprivation on *Vicia faba* nodule NO production (**A**) and nitrate reductase activity (**B**). Data are means ± SD of three biological replicates. Letters above the bars represent significant differences according to one-way ANOVA at *p* < 0.05. NO, nitric oxide; NR, nitrate reductase.

The potential occurrence of Pgb–NO respiration was assessed through the analysis of NO production; total NR activity; and Pgb–NO-respiration-related gene expression (i.e., *Pgb1.1*, *NR1*, and *NR2*). The non-symbiotic Pgb, Pgb1.1, present in all plants, has been shown to be particularly involved in NO metabolism and Pgb–NO respiration [43,45,50]. Previously, using *MtPgb1.1*-over- and under-expressing *Medicago truncatula* lines, Pgb1.1 was shown to finely regulate the level of NO during the symbiotic process and the functioning of Pgb–NO respiration in mature nodules [51]. NR activity and both *MtNR1* and *MtNR2* gene expression have been shown to be related to NO production and Pgb–NO respiration in *M. truncatula* nodules [41,42]. Thus, these three genes were chosen as markers to investigate the involvement of Pgb–NO respiration in faba bean nodules under DS. As reported in Figure 4A, the production of NO increased moderately (40–50%) during the first 4 days of water deficit and then increased by three to four times at 8 and 10 days of DS. Next, we monitored the expression of the *VfPgb1.1*, *VfNR1*, and *VfNR2* genes. As shown in Figure 3C–E, the expression of the three genes increased by two times after 4 days of DS and then decreased after 8 days. This meant that Pgb–NO-respiration-related genes were transiently up-regulated during the first few days of DS. On the other hand, the total NR activity dropped by approximately 65% and by more than 95% after 4 and 8 days of DS, respectively (Figure 4B), which meant that the expression of the genes was not necessarily correlated to the activity of the corresponding enzyme.

Taken together, the increased expression of the *VfAlaAT*, *VfADH*, *VfPgb1.1*, *VfNR1,* and *VfNR2* genes; the increased NO production; and the decreased ATP/ADP ratio after 4 days of DS are all characteristic symptoms of hypoxia. However, after 8 days of DS, the N-fixing capacity dropped sharply (by more than 90%), which suggested that at this stage the nodules were practically no longer functional. Thus, based on the above results, it appears that during DS, the N-fixation capacity of faba bean nodules drops rapidly and that the functioning of SNF could be related to the functioning of Pgb–NO respiration and the maintenance of an energy state compatible with cellular functioning. The question therefore arose of whether Pgb–NO respiration contributes to the maintenance of the energy state of the nodules during DS. To answer this question, we chose to analyze nodules after 4 days of DS. At this stage, the osmolarity of the nodules had increased by 50% (Figure 2A), the N-fixation capacity had only decreased by around 60% (Figure 2B), and the nodules were therefore still functional.

### 2.3. Pgb–NO Respiration Contributes to the Maintenance of Energy State under Osmotic Stress

In the absence of known mutants of NR or phytoglobins in *Vicia faba*, we chose a pharmacological approach to analyze the role of Pgb–NO respiration in the nodules. To this end, we used sodium tungstate (Na_2_WO_4_^=^). WO_4_^=^ is an inhibitor of NR, and more generally of molybdenum (Mo) enzymes, because it substitutes for Mo cofactors as a competitive antagonist, resulting in the inactivation of Mo-dependent enzymes [52,53,54]. However, the absence of irrigation during DS makes it impossible for tungstate (and any other effector) to penetrate the tissues. Thus, to circumvent this obstacle, faba bean plants were first subjected to a 3-day period of water deprivation, and then transferred to a hydroponic culture on a medium containing PEG 6000 at a 43 mM concentration to mimic the 400 mOsm osmotic stress felt by the nodules after 3–4 days of DS (Figure 2A, [55]). Control plants were irrigated for 3 days and then transferred to a hydroponic culture on a medium lacking PEG 6000. PEG-treated (PEG) and control (Ctrl) plants were grown for an additional period of 24 h, in the presence or absence of 4 mM Na_2_WO_4_, before being analyzed.

**Figure 5:**
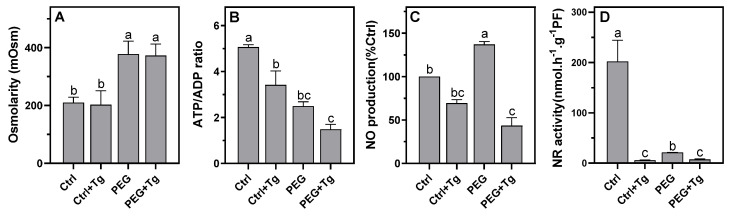
Effects of PEG 6000 osmotic stress and tungstate on *Vicia faba* nodule osmolarity (**A**), ATP/ADP ratio (**B**), NO production (**C**), and nitrate reductase activity (**D**). Data are means ± SD of three biological replicates. Letters above the bars represent significant differences according to one-way ANOVA at *p* < 0.05 (Tukey’s multi-comparison test). NO, nitric oxide; NR, nitrate reductase; PEG, polyethylene glycol 6000; Tg, tungstate.

**Figure 6:**
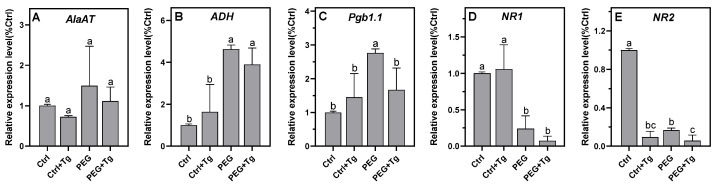
Effects of PEG 6000 osmotic stress and tungstate on the relative expression level of *Vicia faba* nodule genes: alanine aminotransferase (**A**), alcohol dehydrogenase (**B**), phytoglobin1.1 (**C**), nitrate reductase 1 (**D**) and nitrate reductase 2 (**E**). The relative expression of each gene is expressed as the percentage of the control (T = 0) values. Data are means ± SD of three biological replicates. Letters above the bars represent significant differences according to one-way ANOVA at *p* < 0.05 (Tukey’s multi-comparison test). AlaAT, alanine aminotransferase; ADH, alcohol dehydrogenase; Pbg1.1, phytoglobin1.1; NR1, nitrate reductase 1; NR2, nitrate reductase 2; PEG, polyethylene glycol 6000; Tg, tungstate.

At harvest, either in the presence or absence of tungstate, the osmolarity values were around 200 mOsm in the control nodules and 370 mOsm in the PEG-treated nodules (Figure 5A), which was close to the osmolarity values obtained for the nodules cultured on solid soil in the control and after 4 days of DS (Figure 2A). Similar to what was observed in DS nodules, in the presence of PEG 6000 the nodule ATP/ADP ratio decreased by 50%, and NO production increased by 30% as compared to the control (Figure 5B,C). At the gene level, the expression of *VfADH* and *VfAlaAT* increased relative to the control (non-significantly for *VfAlaAT*, Figure 5A,B), indicating that, similarly to what was observed under DS, PEG-6000-treated nodules experienced more intense hypoxia than the control nodules. Interestingly, the osmotic treatment with PEG 6000 resulted in the up-regulation of *VfPgb1.1* expression, as observed at 4 days of DS, but not of *VfNR1* or *VfNR2* expression, which were sharply down-regulated (Figure 6C–E). For its part, the total NR activity dropped by 90%, slightly more than after 4 days of DS. Thus, except for *VfNR1* and *VfNR2* expression, all other parameters analyzed evolved in the same direction between DS and osmotic stress, validating the use of our experimental system on PEG 6000.

The addition of 4 mM sodium tungstate to the culture medium did not alter the osmolarity of the medium (Figure 5A), nor did it alter the expression of hypoxia marker genes (Figure 6A,B). In contrast, in the absence as well as in the presence of PEG 6000, tungstate reduced NO production (Figure 5C) and significantly inhibited *VfNR2* expression and total NR activity (Figure 5D and Figure 6E). Interestingly, in the absence of PEG 6000, the expression of *VfPgb1.1* and *VfNR1* was not modified by tungstate, whereas in the presence of PEG 6000 and tungstate their expression was down-regulated (Figure 6C,D). Finally, in both the control and PEG-6000-incubated nodules, the addition of tungstate led to a reduction in the ATP/ADP ratio (Figure 5B) and thus in the nodule energy metabolism. These results, on the one hand, confirmed that NR inhibition led to the inhibition of Pgb–NO respiration and NO production and, on the other hand, demonstrated that ATP regeneration depended on the functioning of Pgb–NO respiration in both the control and stressed nodules.

## 3. Discussion

As previously observed in faba bean nodules [33], DS was accompanied by an increase in nodule osmolarity and led to a rapid fall in ARA (Figure 2A,B). Under these conditions, it has been shown that SNF inhibition is related to a fall in protein and leghemoglobin [33,56], in conjunction with an increase in proteolysis [56,57]. Furthermore, many studies have shown that in legumes, DS leads to an accumulation of soluble sugars, amino acids, ureides, and different nitrogenous compounds in the nodules [24,29,30,56,58,59]. Thus, regardless of the identification of the signal(s) that initiate(s) the decrease in SNF under DS, it can be concluded that: (1) decreased N_2_-fixation activity is related to the degradation of the machinery (of fixation capacity) and not to the limitation of the supply of substrates (sugars) to operate the machinery; and (2) the export and/or utilization of nitrogen products are more affected by DS than the N_2_-fixation activity. Among the proposed causes of SNF inhibition, we tested the hypothesis that, under the effect of DS, the contraction of nodular structures would increase the O_2_ diffusion barrier of the nodules and enhance the hypoxia that already prevails in unstressed nodules [60]. The decrease in the overall energy status of nodules, as assessed by the ATP/ADP ratio (Figure 2C) and the increase in the expression of the hypoxia marker genes *VfADH* and *VfAlaAT* (Figure 3A,B) supported this hypothesis. This indicated that the increase in the O_2_ diffusion barrier and the consequent decrease in nodule pO_2_ were sufficient to lead to the perception of increased hypoxic stress and the induction of an additional hypoxia response. These results were consistent with the increase in optimal pO_2_ for N_2_-fixation activity observed by [33] in drought-stressed faba bean nodules. Furthermore, they confirmed that, as was already observed under salt stress [34,37,38] (Serraj 1994; Serraj and Drevon 1998; Aridhi 2020), the compaction of nodule structures accentuates the O_2_ diffusion barrier, enhances the hypoxia naturally present in nodules, and reduces nodule energy metabolism. After 8 days of DS, the N-fixing capacity and NR activity dropped by 95% (Figure 2B and Figure 4B), and the nodules turned brown, indicating that their function was strongly impaired. It is likely that at this stage the degradation processes were strongly induced and the DS adaptation processes (transiently induced after 4 days) collapsed. This probably explains why the expression of the genes dropped at this stage and was not observed after 10 days.

The increase in NO production in the nodules during DS (Figure 4A) raised the question of its origin and role in drought-stressed and non-stressed nodules. Previous work has shown that in N_2_-fixing nodules of *M. truncatula* and *M. sativa*, NO is predominantly produced by the tandem action of the NR (which reduces NO_3_^−^ to NO_2_^−^) and mitochondrial electron transfer chain (which reduces NO_2_^−^ to NO) of the plant partner and by the denitrification pathway (NR and nitrite reductase) of the bacterial partner [41,42,61], but that secondary pathways (i.e., NO synthase-like, xanthine dehydrogenase (XDH), NO-forming nitrite reductase (NOFNiR), sulfite oxidase (SOX), or aldehyde oxidase (AO)) could also be involved [43]. The source of drought-induced NO production is still unclear, although evidence suggests the involvement of NR [62] and XDH [63]. In our study, the 30–60% inhibition of NO production (Figure 5C) and 75–95% inhibition of NR activity (Figure 5D) by tungstate suggested that NR is partly involved in NO production. These observations prompt two comments. First, it should be noted that tungstate inhibits Mo enzymes other than NR, such as SOX, AO, NOFNiR, and XDH [52,53,54]. Thus, although RNAi approaches have clearly shown that NR is involved in NO production in *M. truncatula* nodules [41,42] and that *MtNR* genes are significantly more highly expressed than other Mo-enzyme genes in nodules [42], the possibility that the tungstate-dependent inhibition of NO production is linked to the inhibition of one of these enzymes cannot be totally excluded. Second, the functioning of NR implies the presence of NO_3_^−^ in the nodules, but the faba bean plants were grown in a NO_3_^−^-free medium, which raises the question of its origin. The partial inhibition of NO production by tungstate (Figure 5C) indicates that other NO sources, independent of NR and Mo enzymes, were also operating in the nodules. As reviewed in [43], the oxidative synthesis of NO was demonstrated in the nodules of *Lupinus albus, M. truncatula,* and *Glycine max*, presumably through “NOS-like” activity that produced NO and citrulline from arginine. Thus, the arginine-dependent production of NO could enable the regeneration of NO_3_^−^ via Pgb1.1 (Figure 1) and its subsequent supply to NR and Pgb–NO respiration.

In faba bean nodules under DS (Figure 4B) and osmotic stress (Figure 5D), NR activity fell more sharply than in the nodules of other legumes such as *M. sativa*, *Sesbania aculeata*, *Vigna unguiculata*, and *Phaseolus vulgaris*, in which DS caused a transient increase and then decrease in NR activity [64,65,66]. The decrease in NR activity might suggest that it is not involved in NO production, but as it is several orders of magnitude higher than the NO production rate in plants [40], a decrease of 60–80% in its total activity (Figure 4B and Figure 5D) is still more than sufficient to cover the NO synthesis requirements during DS or osmotic stress. Interestingly, the *VfNR1* and *VfNR2* gene expression was up-regulated after 4 days of DS, whereas the NR activity dropped at the same time (Figure 4B). Such an uncoupling between the expression of the NR gene, the amount of NR protein, and the NR activity has already been demonstrated in the leaves of tobacco plants grown on nitrate [67]. Because no other studies have reported on NR gene expression in nodules in response to DS, it is not possible to conclude whether the up-regulation of *VfNR* genes is a general response to DS in all legumes. On the other hand, given that NRs are enzymes that are highly regulated at the post-translational level [68,69] (Lillo 2004; Han 2021), it is not inconsistent that their gene expression is transiently induced in response to hypoxia, as is the expression of *AlaAT*, *ADH*, and *Pgb1.1* genes [70], while its enzymatic activity decreases under water stress, as has been observed in the leaves, roots, and nodules of plants under drought conditions [64,66,71,72]. The presence of two NRs in faba bean raises the question of which of the two isoforms is preferentially involved, if at all, in the production of NO during DS. Indeed, the expression and purification of active fragments of the two Arabidopsis NRs, NIA1 and NIA2, showed that NIA1 exhibits greater NO-forming nitrite reductase activity than NIA2, while the latter exhibits greater nitrate reductase activity [73]. Furthermore, using an RNAi strategy, the authors of [42] also reported that in *M. truncatula* nodules, the production of NO is related to the activity of NR1 rather than NR2. However, the data presented in this study do not allow us to conclude on the involvement of one or the other of the NRs insofar as the two genes are regulated in the same way under DS and under osmotic stress (Figure 3 and Figure 6). One characteristic, however, makes it possible to distinguish the two NRs. In the control conditions in the presence of tungstate, the expression of *VfNR1* was not affected, whereas that of *VfNR2* was significantly repressed (Figure 6D,E). In the leaves of tobacco plants grown in the presence of tungstate, it was shown that *NR* gene expression was deregulated by tungstate, either down- or up-regulated depending on the duration of the treatment [67]. Our results showed that after 1 day of treatment, *NR2* expression was repressed, while that of *NR1* was not modified by tungstate in the control conditions. Although we cannot provide a mechanistic explanation for this differential regulation of *NR* genes, it may be speculated that the maintenance of *NR1* expression in response to excess tungstate is related to its specific role in plant cells (whether or not this is related to the production of NO), allowing the production of Mo-independent and tungstate-insensitive NR with NADH-cytochrome c reductase activity [74]. This hypothesis deserves further investigation in future studies.

Drought-stress-induced NO production has been shown to occur in a wide variety of plants, and the ability of NO to promote adaptive responses to cope with DS is particularly related to its action as an antioxidant and its signaling role in reprogramming plant development and stress response [75,76,77]. In this study, we focused on the metabolic function of NO, as it has been shown to play a role in the regeneration of energy under hypoxia [50,78]. The increase in NO production (Figure 4A) and the up-regulation of *VfPgb1.1*, *VfNR1*, and *VfNR2* expression (Figure 3C–E) at 4 days of DS are strong arguments in favor of the involvement of Pgb–NO respiration in nodule ATP regeneration. Indeed, it has been shown that NO production and the expression of these three genes increase during the development of hypoxic mature nodules [42,51]. Furthermore, the inhibition of NR, and consequently of Pgb–NO respiration, by tungstate (Figure 5D) led to the inhibition of NO production (Figure 5C) and a decrease in the ATP/ADP ratio (Figure 5B) in both the control and stressed nodules. This meant that, in both the control and water/osmotically stressed nodules, at least some of the NO was produced by Pgb–NO respiration, and so Pgb–NO respiration is involved in nodule ATP regeneration. A similar finding was made in M. sativa nodules under salt stress [38]. Thus, taken together, these results confirm our hypothesis that, during both drought and salt stress, the nodule structure compaction, the decrease in O_2_ permeability, and the increase in critical pO_2_ for symbiotic N_2_-fixation [33,36,37] are accompanied by an increase in nodule hypoxia and the involvement of Pgb–NO respiration in cellular energy regeneration.

## 4. Materials and Methods

### 4.1. Biological Material and Growth Conditions

Faba bean (*Vicia Faba* L. minor Bachar) seeds were surface-sterilized by soaking in a 5% sodium hypochlorite solution for 5 min, rinsed 3 times with sterile distilled water, and germinated on agar plates in the dark. Two days later, seedlings were sown into plastic pots (20 cm diameter and 30 cm depth, 2 plants per pot) filled with a mixture of sand and gravel (2:1, *v*/*v*) and watered 3 times per week (120 mL per pot) with nitrate-free nutritive solution (0–15-40: N-P-K) (PLANT-PROD, fertile Inc, Saint Etienne, France). Plants were inoculated 1 week after sowing with FB206 *Rhizobium laguerreae* strain (FB206T JN558651) [47]; FB206 rhizobia were previously grown on yeast-extract mannitol agar (YEMA)-containing plates. Inoculated plates were incubated at 28 °C for 3–4 days [79]. Cells were extracted and pelleted at 13,000 rpm, washed twice, and resuspended in sterile distilled water to a final optical density of 0.01 at 600 nm (OD600) before inoculation. Drought stress (DS) was imposed 30 days after inoculation. In the first set of experiments, plant nodules were harvested after 0, 4, 8, and 10 days of water deprivation. In the second set of experiments, two batches of plants were either water-stressed or well-watered for 3 days. Plants were then transferred to a hydroponic culture on the nutrient medium previously used for watering in the absence (control) or presence (osmotic stress) of 43 mM PEG 6000. Four mM sodium tungstate (Na_2_WO_4_) was added to the culture medium of half of the control and half of the osmotically stressed plants. The culture media were bubbled with air to oxygenate them. After 24 h of culture, the nodules were harvested. At least three independent biological replicates were arranged in a completely randomized design and were conducted in a growth room under controlled conditions (temperature of 22–25 °C) with a 16 h light/8 h dark photocycle. For both DS and osmotic-stress experiments, harvested nodules were either immediately processed for NO production and nitrogen-fixing capacity quantification, or frozen in liquid nitrogen and stored at −80 °C for further analysis.

### 4.2. Nitrogen-Fixing Capacity Measurement

The nitrogenase activity of the nodules was determined in vivo by measuring the acetylene-reducing activity (ARA, [80]). Nodulated roots were harvested and incubated at 30 °C for 1 h in rubber-capped tubes containing 10% acetylene atmosphere. Ethylene concentrations were determined by gas chromatography (Agilent GC 6890N, Agilent Technologies, Les Ulis, France) using a GS-Alumina separating capillary column.

### 4.3. Measurement of NO Production

NO detection was performed as in [41] using a Cu (II) fluorescein (CuFL) probe (Strem Chemicals, Bischheim, France) with the following changes: 20 to 30 mg of detached fresh nodules were incubated in the dark at 23 °C in 1 mL of detection buffer (10 mM Tris-HCl pH 7.4, 10 mM KCl) and in the presence of 10 µM CuFL probe. The production of NO was measured for 1 h with a spectrofluorometer/luminometer (Xenius, SAFAS, Monte Carlo, Monaco).

### 4.4. Osmolarity of Nodule Cells

About 50–100 mg of frozen dry nodules was crushed into a powder in liquid nitrogen and transferred in Eppendorf tubes. After thawing, samples were centrifuged for 30 min at 20,000× *g*. Of the nodule supernatant extract, 10–20 µL was taken and diluted to 100 µL in an Eppendorf tube for osmolarity determination. Osmolarity was measured with a precalibrated micro-osmometer Roebling 1-DR (Fischer Scientific, Illkirch, France).

### 4.5. Extraction and Measurement of Adenine Nucleotides

Adenine nucleotides were extracted essentially as in [41]. All extraction steps were carried out at 4 °C. Frozen material (40–60 mg) was crushed in liquid nitrogen with 300 mL of perchloric acid solution, containing 7% (*v*/*v*) HClO_4_ and 25 mM Na_2_EDTA, with a mortar and pestle. After thawing, the extract was collected and the mortar was rinsed with 200 mL perchloric acid solution, which was then pooled with the extract. The sample was centrifuged for 5 min at 13,000× *g*. The supernatant was quickly and carefully neutralized at pH 5.6–6.0 using a 2 M KOH–0.3 M MOPS solution. KClO_4_ precipitate was discarded by centrifugation (5 min, 13,000× *g*). Adenine nucleotides of the supernatant were measured in a Xenius spectrofluorometer/luminometer (Xenius, Safas, Monaco) using the ATPlite one-step assay system (ATPLT1STP-0509; Perkin-Elmer, Villebon-sur-Yvette, France) according to the manufacturer’s instructions.

### 4.6. Measurement of NR Activity

Tissue samples were ground with a mortar and pestle in liquid nitrogen. The total proteins were extracted from 100 mg of powder using the following extraction buffer: 25 mM Tris-HCl, pH 8.5; 1 mM EDTA; 20 μM FAD; 0.04% Triton X100; 10 μM NaMO_4_; 1 mM DTT; 20 µM L-trans-epoxysuccinyl-leucylamido-[4-guanidino] butane (E64); and 2 mM phenylmethylsulfonyl fluoride (PMSF). The extracts were then centrifuged (15,000× *g*, 15 min). The NR activity was measured by quantifying the NO_2_^−^ produced in the reaction mixture containing: the enzyme extract in 0.2 M HEPES, pH7.0; 15 mM KNO_3_; and 250 μM of NADH [81]. The reaction was stopped after 30 min by boiling the samples for 3 min at 100 °C. The nitrite produced was measured using Griess reagent (1% (*w*/*v*) sulphanilamide in 1 M HCl and 0.01% (*w*/*v*) NEDD (N-1-naphthylethylenediamine dihydrochloride) in water). After incubation for 30 min at ambient temperature, samples were centrifuged for 10 min at 13,000× *g*, and the absorbance of the supernatant was read at 540 nm. Assay blanks contained enzymatic extracts boiled at 100 °C for 3 min before the addition of KNO_3_ and NADH.

### 4.7. RNA Isolation, Reverse Transcription, and Gene Expression

RNAs were isolated from 100 mg of frozen material ground in liquid N2 using the Monarch Total RNA Kit, following the manufacturer’s instructions (New England BioLabs, Evry-Courcouronnes, France). RNA quality and quantity were checked with a nanodrop (Thermo Fisher NanoDrop2000, Illkirch, France) and electrophoresis gel. DNase treatment was carried out before the synthesis of the cDNAs by GoScript reverse transcriptase (Promega, Charbonnières-les-Bains, France). The RT-qPCR was conducted using a Go-Taq qPCR master mix kit according to manufacturer’s instructions (Promega, Charbonnières-les-Bains, France). Sequences of *VfNR2*, *VfPgb1.1*, *VfADH1*, and *VfAlaAT* genes were identified in the Pulse Crop Database (PCD; https://www.pulsedb.org, copyright © 2022–2022) using the Blast tool with the sequence of the *M. truncatula* orthologue gene. The sequence of the *VfNR1* gene was identified thanks to the recent release of the high-quality chromosome-scale assembly of the faba bean genome ([82]; https://www.biorxiv.org/content/10.1101/2022.09.23.509015v1, 23 September 2022). A search by Blast of the Hedin-variety fava bean genome (www.fabagenome.dk, revised 14 March 2022) was conducted to identify the *VfNR1* gene. *Vicia faba Pgb1.1*, *NR1*, and *NR2* genes were identified to be the orthologues of the *M. truncatula Pgb1.1*, *NR1*, and *NR2* genes and named accordingly. The expression of the different genes was normalized with two housekeeping genes, *ACT1* and *ELF1A* [83]. RT-qPCR analyses were carried out in triplicate, using the primers reported in Table 1.

### 4.8. Statistical Analysis

Statistical analyses were performed using multivariate analysis and two-way ANOVA followed by a Tukey test in RStudio software (version 2022.07.1–554, Rstudio, Boston, MA, USA) and GraphPad Prism8 software (version 8.0.2, Graphpad Software, San Diego, CA, USA). Data were considered as significantly different when *p ≤* 0.05. RT-qPCR data analysis was carried out using RqPCRBase, an R package working in the R computing environment for the analysis of quantitative real-time PCR data [84].

## 5. Conclusions

In this work, we hypothesized that, under drought stress, faba bean nodules experience reinforced hypoxia, and that Pgb–NO respiration could be involved in maintaining nodule energy status. Our data showed that: (1) the drought-stressed and osmotically stressed nodules, like salt-stressed nodules, were subject to more intense hypoxia than the control nodules and (2) the stressed-nodule production of NO transiently increased and partly contributed via Pgb–NO respiration to the maintenance of the energetic state of the nodule.

## Figures and Tables

**Table 1:** Primers used for qRT-PCR analysis.

Gene	Accession ID	Description	Forward and Reverse Primer (5′–3′)	Efficiency (%)	Reference
*ACT1* ^(1)^	P30164	actin 1	F: GCTGTCCTCTCCCTCTATGCA R: GCCGAGGTGGTGAACATATACC	92.0	[83]
*ELF1A* ^(1)^	AJ222579	eukariotic elongation factor 1-alpha	F: GTGAAGCCCGGTATGCTTGT R: CTTGAGATCCTTGACTGCAACATT	100.5	[83]
*Pgb1.1*	CSVX01042149.1	phytoglobin 1.1	F: ACTTGAGAGCTAGTTCTGCAGAA R: TGTTTCACGGAAGAGCAAGAAG	99.6	This work
*NR1*	Vfaba.Hedin2.R1.2g082320	nitrate reductase 1	F: AGCTTGGTTCTATAAACCGGAG R: CTGAGTAGTCTCTGAGTCAACC	98.3	This work
*NR2*	CSVX01059671.1	nitrate reductase 2	F: TAGTTTGCGCTGGTAACCGT R: ACCGAATTTGAAACCGCTGC	102.8	This work
*AlaAT*	CSVX01002777.1	alanine aminotransferase	F: CGCCACAGGAATCGTTGTTG R: CAAACGGGTGACAATGGCTG	90.4	This work
*ADH*	CSVX01017814.1	alcohol dehydrogenase	F: ACACCCTCACCTACACTCTC R: TCAAGATACTCTTCACCTCCCT	101.43	This work

^(1)^ Housekeeping genes.

## Data Availability

Not applicable.

## Abbreviations

ARA	acetylene-reducing activity
CuFL	Cu (II) fluorescein
DAF-2	4,5-diaminofluorescein
NO	nitric oxide
NR	nitrate reductase
Pgb	phytoglobin
SNF	symbiotic nitrogen fixation
